# Involvement of Proline Oxidase (PutA) in Programmed Cell Death of *Xanthomonas*

**DOI:** 10.1371/journal.pone.0096423

**Published:** 2014-05-01

**Authors:** Surbhi Wadhawan, Satyendra Gautam, Arun Sharma

**Affiliations:** Food Technology Division, Bhabha Atomic Research Centre, Mumbai, India; University of Nebraska-Lincoln, United States of America

## Abstract

*Xanthomonas campestris* strains have been reported to undergo programmed cell death (PCD) in a protein rich medium. Protein hydrolysates used in media such as nutrient broth comprise of casein digest with abundance of proline and glutamate. In the current study, *X. campestris* pv. *campestris* (Xcc) cells displayed PCD when grown in PCD inducing medium (PIM) containing casein tryptic digest. This PCD was also observed in PCD non-inducing carbohydrate rich medium (PNIM) fortified with either proline or proline along with glutamate. Surprisingly, no PCD was noticed in PNIM fortified with glutamate alone. Differential role of proline or glutamate in inducing PCD in Xcc cells growing in PNIM was studied. It was found that an intermediate product of this oxidation was involved in initiation of PCD. Proline oxidase also called as proline utilization A (PutA), catalyzes the two step oxidation of proline to glutamate. Interestingly, higher PutA activity was noticed in cells growing in PIM, and PCD was found to be inhibited by tetrahydro-2-furoic acid, a competitive inhibitor of this enzyme. Further, PCD was abolished in Xcc Δ*putA* strain generated using a pKNOCK suicide plasmid, and restored in Xcc Δ*putA* strain carrying functional PutA in a plasmid vector. *Xanthomonas* cells growing in PIM also displayed increased generation of ROS, as well as cell filamentation (a probable indication of SOS response). These filamented cells also displayed enhanced caspase-3-like activity during *in situ* labeling using a fluorescent tagged caspase-3 inhibitor (FITC-DEVD-FMK). The extent of PCD associated markers such as DNA damage, phosphatidylserine externalization and membrane depolarization were found to be significantly enhanced in wild type cells, but drastically reduced in Xcc Δ*putA* cells. These findings thus establish the role of PutA mediated proline oxidation in regulating death in stressed *Xanthomonas* cells.

## Introduction

*Xanthomonas campestris* pv. *glycines* (Xcg), a pathogen of soybean causing bacterial pustule disease, and other *Xanthomonas campestris* pathogenic strains [namely *X. campestris* NCIM 2961 and *X. campestris* pv. *malvacearum*] were earlier reported in this laboratory to undergo growth medium dependent programmed cell death (PCD) during post-exponential phase [1], [2], [3], [4], [5]. The process was later found to be associated with an increase in NADH generation leading to formation of reactive oxygen species (ROS) [4], [6]. Since the PCD process in this bacterium was observed to be induced under conditions where the protein content of the medium was higher than the carbohydrate content, a fine balance of these two major nutrients was thought to be an essential factor governing metabolism associated survival of this microorganism in laboratory culture conditions. The designed conditions of growth of this organism in laboratory are quite distinct from its natural habitat on soybean leaf, where it is more accustomed to carbohydrate rich environment. As *Xanthomonas* represents one of the major groups of bacterial plant pathogens, understanding the balance between its survival and death could not only have broad practical significance in agriculture but also provide significant clues to microbial growth control and regulation. Casein digest is one of the PCD inducing constituents of PIM for *Xanthomonas* and predominantly provides very high levels of glutamate and proline. Hence, the effect on PCD process of *Xanthomonas campestris* pv. *campestris* (Xcc) upon addition of these two amino acids, either individually or in combination in PCD non-inducing medium (PNIM), was examined. The study further focused on the activity of one of the major enzymes, PutA (proline utilization A, also called proline oxidase or proline dehydrogenase) which is involved in the metabolism of proline in bacteria, including Xcc.

PutA is located in the bacterial membrane (or inner mitochondrial membrane in higher organisms). Contrary to eukaryotes where proline oxidation is carried out by two separate enzymes, proline oxidase (POX) and P5C (pyrroline-5-carboxylate) dehydrogenase (P5CDH), in bacteria, PutA contains both these activities in a single protein [7]. POX converts proline to P5C, which is non-enzymatically hydrolyzed to γ-glutamate semialdehyde, and further oxidized by P5C dehydrogenase to glutamate [8].


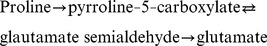
(1)

Glutamate can be converted to α-ketoglutarate through deamination, which may be incorporated into the tricarboxylic acid (TCA) cycle. Conversion of glutamate back to proline involves three enzymatic steps, with the initial two steps catalyzed by the bifunctional enzyme P5C synthase to generate P5C, which is subsequently reduced to proline by the NADPH-dependent Pyrroline-5-carboxylate reductase (PYCR) [8].

In the current study to understand the role of proline metabolism in PCD of *Xanthomonas, putA* gene was knocked out from one of the pathogenic strains of *Xanthomonas*, namely *X*. *campestris* pv. *campestris* strain 8004 (Xcc 8004). The wild type and mutant strain were examined under similar growth conditions for viability, as well as PCD specific markers such as activity of caspase-3 like protease, level of phosphatidylserine (PS) externalization and the extent of DNA damage. For further understanding, the intracellular reactive oxygen species (ROS) level as well as the change in membrane potential was analyzed. Additionally, the *putA* gene was cloned in an *E.coli - Xanthomonas* shuttle vector, and Xcc 8004 Δ*putA* was complemented for PutA activity by transforming it with the recombinant plasmid, and the above mentioned biochemical and molecular markers were examined.

## Materials and Methods

### Bacterial Strains and Growth Conditions

*Xanthomonas* strains were grown at 26±2°C in a rotary shaker at 150 rpm in Luria-Bertani (LB) broth {PCD inducing medium (PIM)}, or starch broth (SB) {PCD non-inducing medium (PNIM); 1% starch, 0.3% K_2_HPO4.3H_2_O, 0.15% KH_2_PO4, 0.2% ammonium sulphate, 0.05% L-methionine, 0.025% nicotinic acid, and 0.025% L-glutamate, pH 6.8±0.2}. All *E.coli* strains were grown in LB medium on a rotary shaker (150 rpm) at 37±2°C. The cell number was enumerated by the standard plate count method [1].

### Chemicals

Antibiotics (kanamycin and gentamycin), ninhydrin, tetrahydro-2-furoic acid (THFA) and 2′, 7′-dichlorohydrofluorescein-diaceate (H_2_DCFDA) were purchased from Sigma (St. Louis, MO). Proline was purchased from SRL (India). Sulphosalicylic acid, LB media and salts were purchased from Himedia, India. Restriction enzymes and DNA ligation kit were purchased from New England Biolabs (NEB, USA) and Fermentas (USA) respectively. Pfu polymerase was purchased from Stratagene (Agilent, USA).

### Determination of Intracellular Level of Proline in *Xanthomonas* Cells

Intracellular proline levels were determined in Xcc cells as mentioned before [9]. Briefly, an aliquot of overnight grown culture was washed twice with equal volume of PBS (Phosphate buffer saline) (10 mM, pH 7.5) and resuspended in 3% sulphosalicylic acid. The cells were sonicated for 2 min (60% power) followed by heating at 95°C for 10 min. The culture was centrifuged at 12,000×g for 10 min. To this clear supernatant 1 ml glacial acetic acid and 1 ml acidic ninhydrin (prepared by warming 1.25 g ninhydrin in 30 ml glacial acetic acid and 20 ml 6 M phosphoric acid) was added. This reaction mixture was kept at 100°C for 1 h after which the reaction was terminated on ice bath for 20 min. The reaction mixture was extracted with 2 ml toluene and the absorbance was read at 520 nm using UV–visible spectrophotometer (UV4, Unicam, Cambridge, UK).

### Estimation of Intracellular Cysteine Levels

The intracellular cysteine levels were estimated as described earlier [10]. Briefly, overnight grown cells were washed twice with PBS (10 mM, pH 7.5) and resuspended in 5% perchloric acid. Samples were boiled for 10 min followed by centrifugation at 12,000×g for 10 minutes. The clear supernatant (100 µl) was mixed with 100 µl acetic acid and 100 µl acidic ninhydrin reagent (prepared by mixing 250 mg ninhydrin in 6 ml acetic acid and 4 ml concentrated HCl and kept at 100°C for 15 min). The reaction mixture was cooled on ice and diluted to 1 ml with 95% ethanol. The absorbance was read at 560 nm using UV–visible spectrophotometer.

### Measurement of PutA Activity in Terms of Proline Oxidase Activity

The proline oxidase activity was assayed according to Dendinger and Brill (1970) [11]. Briefly, an aliquot of 24 h culture was washed twice with PBS (10 mM, pH 7.5) and resuspended in 100 mM Tris-HCl (pH 7.4). Wherever required, inhibitors were added to the cell suspension and incubated at room temperature for 30 min. For permeabilization, 5 µl toluene was added to the cell suspension. After 10 min, 1 ml L-proline (1 M) and 200 µl o-aminobenzaldehyde (50 mM in 20% ethanol) was added. The reaction mixture was kept for shaking at 26±2°C for an hour and was terminated by adding 200 µl trichloroacetic acid (20%). The cell debris was removed by centrifugation at 12,500×g for 15 min. The absorbance of the clear supernatant was measured at 443 nm using UV - visible spectrophotometer. The millimolar extinction coefficient of the P5C (pyrroline-5-carboxylate) - o-aminobenzaldehyde complex is 2.71 [11]. PutA activity was expressed as micromoles of P5C formed min^−1 ^mg^−1^ protein. The protein content was estimated by Lowry’s method [12].

### Construction of *putA* Knockout in *Xanthomonas*

To further verify the role of PutA in metabolic stress induced PCD of *Xanthomonas*, a *putA* knockout of *Xanthomonas campestris* pv. *campestris* strain 8004 (Xcc 8004) was constructed by insertional mutagenesis using pKNOCK-Km suicide plasmid (2 kbp) vector which has R6Kγ origin of replication [13]. Hence, the plasmid can only replicate in only those *E. coli* strains which can provide the replication initiator pi protein [14]. Xcc was used for this study because its genome sequence is known and it also shows post exponential cell death in LB medium similar to Xcg. An internal 600 bp region of *putA* gene (complete size around 3.2 kbp) was amplified using FP1 and RP1 primers (Table 1). Hind III restriction enzyme site was introduced at each end. The derivative pKNOCK plasmid carrying the 600 bp *putA* gene fragment is henceforth termed as pKNOCK-putA. This pKNOCK-putA plasmid was then used to transform competent *E. coli* PIR1 cells (prepared using CaCl_2_ method) by heat shock and transformants were selected on LB-kanamycin (25 µg ml^−1^) - agar plate. Competent Xcc cells (prepared by washing thrice with 10% ice-chilled glycerol) were transformed using electroporation [15]. pKNOCK plasmid disrupts the target gene by insertional mutagenesis (Fig. S1). This is achieved by homologous recombination between the target gene and the complimentary gene fragment cloned in the pKNOCK plasmid. The integration of pKNOCK-putA into the *putA* gene was confirmed by PCR amplification of full length *putA* gene from the transformed Xcc colony.

**Table 1:** Bacterial strains, plasmids and primers used in this study.

Strain/plasmid/primer	Relevant characteristic	Source
*Xanthomonas*		
*Xanthomonas campestris* *campestris* str 8004 (Xcc)	Wild type; Rif^r^	[60]
Xcc Δ*putA*	*putA* deletion mutant of Xcc 8004; Rif^r^ Kan^r^	This work
Xcc Δ*putA/*pPutA	Xcc Δ*putA* harboring pBBR1MCS5 containing the entire *putA* gene;; Rif^r^ Kan^r^ Gm^r^	This work
*E. coli*		
*E. coli* pir1	{F- Δlac169 rpoS(Am) robA1 creC510 hsdR514 endA recA1 uidA(ΔMluI)::pir-116}	Invitrogen
*E. coli* pir1/pKNOCK-putA	*E. coli* pir1harboring suicide plasmid pKNOCK-putA; Kan^r^	This work
*E. coli* DH5α	F– Φ80*lac*ZΔM15 Δ (*lac*ZYA-*arg*F) U169 *rec*A1 *end*A1 *hsd*R17 (rK–, mK+) *pho*A *sup*E44 λ– *thi*-1 *gyr*A96 *rel*A1	Invitrogen
*E. coli* DH5α/pPutA	*E. coli* DH5α harboring pBBR1MCS5 containing the entire *putA* gene; Gm^r^	This work
Plasmids		
pKNOCK-Km	Suicide vector in *Xanthomonas;* Kan^r^	[13]
pKNOCK-putA	pKNOCK-Km with an internal gene fragment of *putA*	This work
pBBR1MCS-5	Broad host range cloning vector; Gm^r^	[16]
pPutA	pBBR1MCS5 containing the entire *putA* gene; Gm^r^	This work
Primers		
FP1	5′ CCGAAGCTTATGTGCGTGGCCGAAGCCTTGC 3′	This work
RP1	5′ CCGAAGCTTCTTGGCCAGTTGTGCCAGCTCC 3′	This work
FP2	5′ CCCAAGCTTGTCCCAACCCCTTCGGACA 3′	This work
RP2	5′ CGCGGATCCTCAGTCACCCAAGGTCAG 3′	This work

### Cloning of Xcc *putA* in a Broad Host Range (bhr) Shuttle Vector and Complementation of Xcc Δ*putA* Strain

The *putA* gene in Xcc 8004 is present in single copy. It is flanked upstream by a gene for hypothetical protein (XC_3906; location: 4,610,064–4,610,495) and downstream by IS1478 transposase gene (XC_3908; location: 4,614,266–4,615,633) (Fig. S2A). The full length *putA* gene excluding the promoter region in Xcc 8004 is 3.2 kb in size (XC_3907; location: 4,610,819–4,614,019). For complementing Xcc Δ*putA* strain with functional PutA, the 319 bp non-coding region present between XC_3906 gene and *putA* was amplified along with the *putA* gene using Pfu polymerase, FP2 and RP2 primers (Table 1) by colony PCR technique. This non-coding 319 bp sequence contains the *putA* promoter region which has not been characterized yet. In this study, BPROM software was used to identify the possible promoter region of *putA* and the findings have been shown in Fig. S2B. The PCR product (3.52 kb in size) was cloned into a broad host range (bhr) vector pBBR1MCS5 (Fig. S3). This vector was originally derived from pBR322 by subsequent modifications to have several advantages such as relatively smaller size (4.7 kb), extended multiple cloning site (MCS), possibility of direct selection of recombinant plasmid in *E.coli* via disruption of the LacZα peptide, mobilizable when the RK2 transfer functions are provided in *trans,* and compatible with IncP, IncQ and IncW group plasmids, as well as with ColE1 and P15a-based replicons [16]. The recombinant plasmid carrying *putA* gene is henceforth termed as pPutA and was used to transform *E.coli* DH5α cells. The transformants were selected on LB-gentamycin plate (10 µg ml^−1^). Xcc Δ*putA* strain was further transformed with pPutA by electroporation as described above.

### Assay of Caspase-3-like Activity

Caspase-3-like activity was assayed according to the manufacturer’s guidelines (caspase-3 assay kit, BD Pharmingen, USA). Briefly, a 1 ml aliquot of 24 h grown culture was washed twice with phosphate buffered saline (PBS) (10 mM, pH 7.5) and resuspended in saline (0.85%). The cell suspension was centrifuged at 12,500×g for 10 min. The pellet was resuspended in 100 µl of sodium phosphate buffer (10 mM, pH 7.5), mixed with 1 ml cell lysis buffer {Tris-HCl (10 mM), sodium phosphate buffer (10 mM, pH 7.5), NaCl (130 mM), triton X-100 (1%) and sodium pyrophosphate (10 mM)} and kept at 4°C for 4 h for lysis. The cell lysate was then centrifuged at 12,500×g for 15 min and an aliquot (50 µl) of the above supernatant was used for caspase-3 assay using synthetic fluorogenic substrate Ac-DEVD-AMC (BD Pharmingen, USA) as described earlier [2].

### Analysis of Active Caspase-3-like Protein *in situ* by FITC-DEVD-FMK Staining

The assay was carried out using caspase-3 detection kit (Catalog no. QIA91, Calbiochem). An aliquot (250 µl) of 24 h grown cell culture containing ∼10^6^ cfu ml^−1^ was washed twice with PBS (10 mM, pH 7.5). The cell pellet was resuspended in 300 µl PBS. To this cell suspension 1 µl of FITC-DEVD-FMK was added and incubated at room temperature for 30 min in dark. After that, the cells were centrifuged at 12,500×g for 5 min and supernatant was discarded. The cells were washed twice with wash buffer and resuspended in 200 µl of the same. An aliquot (10 µl) was smeared on a glass slide, air dried and examined under a fluorescent microscope (Carl Zeiss, Germany) using oil immersion objective (100x) and filter set 9 (Carl Zeiss, Germany; Excitation: 450 nm; emission: 515 nm).

### Observation of Cell Filamentation

An aliquot (1 ml) of cells grown in PIM (24 h) or PNIM (72 h) was washed twice with PBS (10 mM, pH 7.5), resuspended in saline (0.85%). An aliquot (10 µl) was smeared on a glass slide, air dried, heat fixed, stained with crystal violet and examined under a microscope (Carl Zeiss, Germany) using oil immersion objective (100X) for observation of cell filaments.

### Quantification of DNA Damage by TUNEL (Terminal deoxynucleotidyl transferase dUTP Nick End Labeling) Assay

TUNEL assay was performed using the APO-Direct kit, BD Pharmingen as described earlier [17]. Briefly, an aliquot (1 ml) of 24 h grown cell culture containing ∼10^6^ cfu ml^−1^ was washed twice with PBS (10 mM, pH 7.5). The cell pellet was resuspended in 50 µl DNA labeling solution [reaction buffer (10 µl), Terminal deoxynucleotidyl transferase (TdT) enzyme (0.75 µl), FITC-dUTP (8 µl) and distilled water (32.25 µl)] and incubated for 60 min at 37°C in dark. After that, 1 ml rinse buffer was added and cell suspension was centrifuged at 12,000×g for 10 min. This rinsing step was repeated once more. PI/RNase staining buffer (500 µl) was added to the samples which were further incubated in dark for 30 min, and analyzed by Fluorescence Activated Cell Sorter (FACS) (10^5^ cells for each sample) using flow cytometry system (Partec CyFlow space, Germany).

### Quantification of Phosphatidylserine (PS) Externalization Using Annexin-V Labeling

The assay was performed using annexinV-FITC apoptosis detection Kit (catalog no. 556547, B D Pharmingen) as described earlier [17]. Briefly, an aliquot (1 ml) of 24 h cell culture containing ∼10^6^ cfu ml^−1^ was washed twice with PBS (10 mM, pH 7.5) and the pellet was resuspended in 250 µl of the same buffer. An aliquot (650 µl) of annexinV binding buffer (10 mM HEPES, pH 7.4; 140 mM NaCl and 2.5 mM CaCl_2_) and annexinV (5 µl) were added to the cell suspension and incubated in dark for 15 min. Propidium iodide (5 µl, 50 µg ml^−1^) was then added into the cell suspension and incubated at ambient temperature in dark for 15 min. For each analysis, 10^5^ cells were analyzed by flow cytometry (Partec CyFlow space, Germany). Data was analyzed using FCS Express V4 software (demo version).

### Analysis of Reactive Oxygen Species (ROS) Generation by Dichlorohydrofluorescein Staining

Dichlorohydrofluorescein (H_2_DCFDA) staining was carried out as mentioned previously [4]. Briefly, *Xanthomonas* cells were grown at 26±2°C in a rotary shaker at 150 rpm in culture medium (LB) for 18 h. A 2 ml aliquot was withdrawn and centrifuged at 12,500×g for 2 min and the pellet was resuspended in 1 ml saline (0.85%). It was then incubated with 2 µl H_2_DCFDA (5 mM, prepared in absolute ethyl alcohol) at room temperature for 30 min. An aliquot was smeared on a glass slide, air dried and examined under a fluorescent microscope (Carl Zeiss, Germany) using oil immersion objective (100x) and filter set 15 (Carl Zeiss, Germany; excitation: 546 nm; emission: 590 nm).

### Determination of Membrane Potential

It was carried out using BacLight bacterial membrane potential assay kit (Molecular Probes, catalog no. B34950) as per the manufacturer’s guidelines. Briefly, an aliquot (1 ml) of 24 h grown cell culture containing ∼10^6^ cfu ml^−1^ was washed twice with PBS (10 mM, pH 7.5) and the pellet was resuspended in 1 ml of the same buffer. For preparing the depolarized control sample, 10 µl of an uncoupler CCCP (carbonyl cyanide 3-chlorophenylhydrazone) (50 mM) was added to 1 ml of cell suspension (∼10^6^ cfu ml^−1^) and incubated at room temperature for 30 min. CCCP is a proton ionophore which dissipates membrane potential by eliminating the proton gradient. Thereafter, cells were stained with 5 µl (50 nM final concentration) of DiOC_2_(3) (3,3′-diethyloxacarbocyanine iodide), and 10^5^ cells were analyzed by flow cytometry (Partec CyFlow space, Germany, and Express V4 software, demo version).

### Statistical Analysis

The experiments were repeated in three independent sets, each set comprising of three replicates. The mean and standard deviations (SD) were calculated taking all the data points in consideration. The mean values were further compared using one-way ANOVA (analysis of variance) test for establishing the significance of variation among the means (p<0.05). With respect to flow cytometry analysis and microscopic examinations, a representative data is presented.

## Results

### Addition of Proline but not Glutamate in PNIM Induced PCD

**Figure 1:**
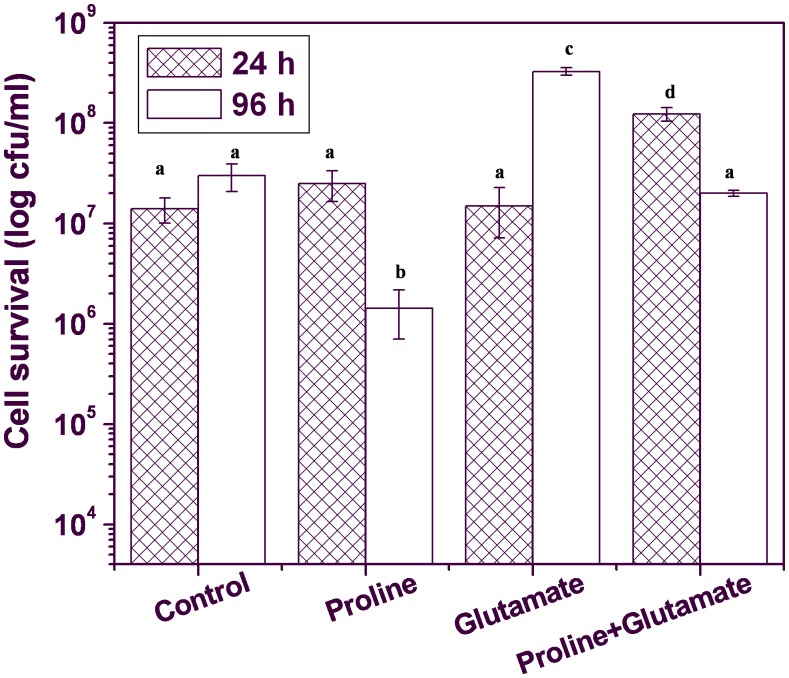
The effect of proline and glutamate supplementation on the survival of *Xanthomonas campestris* pv. *campestris* in PNIM. Different letters on the bars indicate that the means are significantly different at p<0.05.

Glutamate and proline are the two major casein amino acids present in Luria Bertani (LB) broth [18]. Hence, the effect of these amino acids (100 mM concentration) either individually or in combination was assessed on the cells growing in PCD non-inducing medium (PNIM). When *Xanthomonas* cells were grown in PNIM supplemented with proline (100 mM), the cell count peaked at 24 h of incubation followed by a sharp decline in viable cell number during further post-exponential incubation under similar growth conditions (150 rpm, 26°C) (Fig. 1). At the end of 96 h of incubation, one log cycle decrease in viable cell count was observed. The cell number was found to be more in the cultures supplemented with glutamate with respect to control cells (without glutamate) and it remained almost unchanged at ∼10^8^ cfu/ml at the end of 96 h of incubation (Fig. 1). Interestingly, the growth profile upon addition of both proline and glutamate together in the culture medium (PNIM) behaved similar to proline addition and the loss of viability by around one log cycle was noticed at 96 h. The observations thus indicated a regulatory role of proline in death of *Xanthomonas* cells under unfavorable nutritional condition.

### Enhanced Puta Activity Observed during PCD in PIM

**Figure 2:**
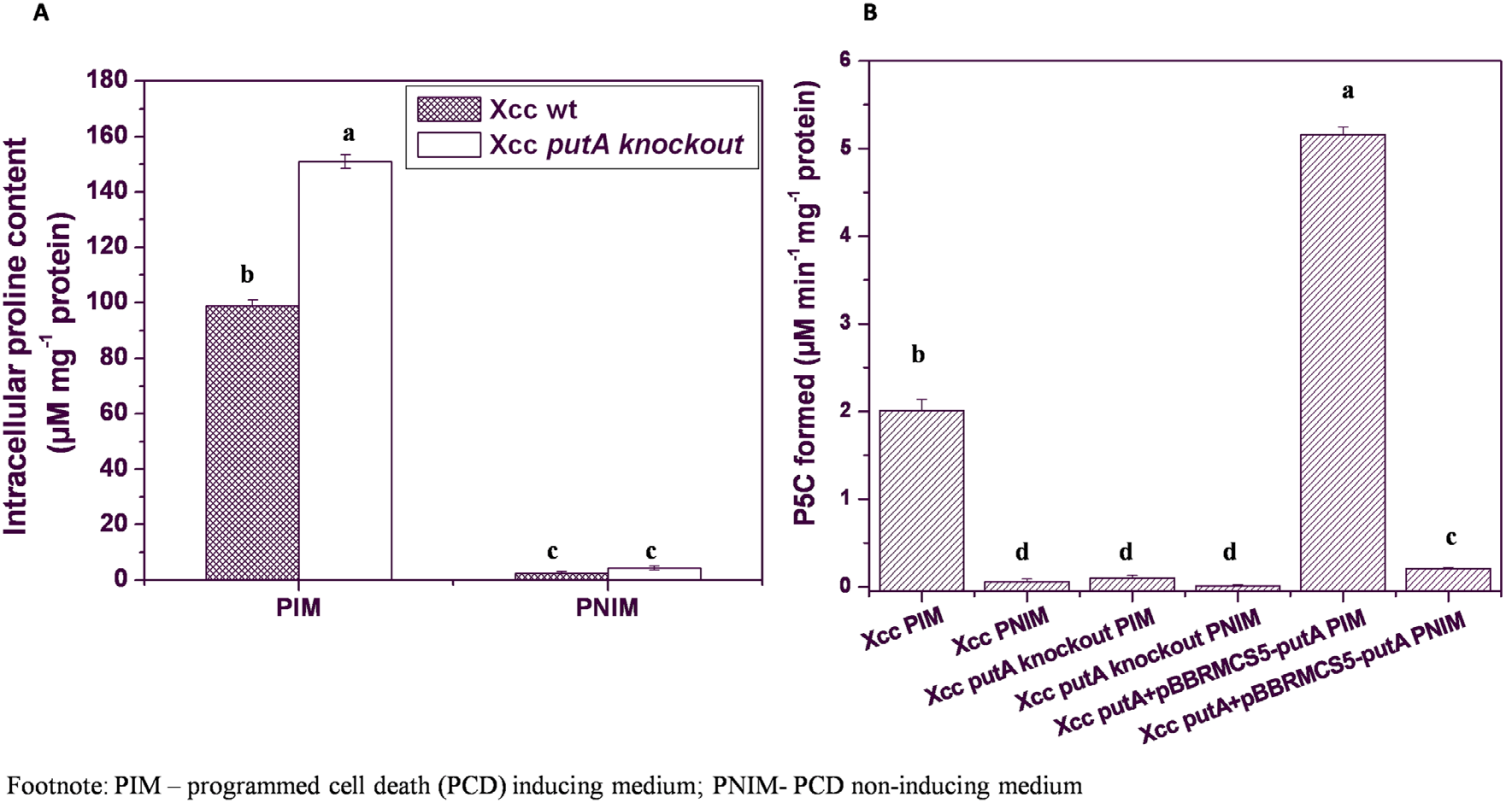
Status of intracellular proline levels and PutA activity in Xcc strains. (**A**) Intracellular proline level, and (**B**) PutA activity in Xcc 8004, Xcc Δ*putA* and Xcc Δ*putA-*pPutA cells in PIM and PNIM. Different letters on the bars indicate that the means are significantly different at p<0.05.

The above observations indicated the role of proline in induction of PCD in this bacterium. The growth medium favoring PCD during this study namely LB medium also contains higher concentration of proline compared to other amino acids [18], [19]. Its concentration in casein tryptic digest is close to 6%, next to glutamic acid (around 15%), which can also get converted to proline enzymatically inside the cell. As expected, intracellular proline level in PNIM was found to be quite low (2.4 µM mg^−1^ protein) in Xcc which increased to around 35 fold (83 µM mg^−1^ protein) in PIM growing cells (Fig. 2A). Increased substrate concentration might affect the activity of respective enzyme eventually. Hence, examining the status of the enzyme, proline oxidase (PutA) involved in metabolism of proline becomes important to further understand the regulation of PCD in these cells.

Unlike eukaryotes, bacterial PutA has dual activity, where first proline oxidase (or dehydrogenase) activity oxidizes proline to P5C (pyrroline-5-carboxylate), which spontaneously converts to γ-glutamate semialdehyde [7], [20]. This γ-glutamate semialdehyde is then eventually oxidized to glutamate by P5C dehydrogenase activity of PutA [7]. In this study the PutA activity was measured as its proline oxidase (POX) activity. In Xcc cell lysates, PutA activity was monitored in terms of P5C product formation by measuring the adduct formed between P5C and o-aminobenzaldehyde because the formation of P5C is very specific to this pathway. PutA activity was found to be 0.06 µM min^−1 ^mg^−1^ protein in PNIM in Xcc wt cells which increased by around 34 fold to 2.01 µM min^−1 ^mg^−1^ protein in PIM (Fig. 2B). This observation can be attributed to the higher intracellular proline found in Xcc cells cultured in PIM. It has been reported that L-proline is preferentially used as a carbon source by *E.coli* growing in LB medium and this amino acid gets depleted quite early during its growth [18]. Intracellular level of cysteine was also checked as a control. Basal level of cysteine was found to be comparatively high (50 µM mg^−1^ protein) even in PNIM indicating its constitutive requirement for cellular metabolism. However, in PIM growing Xcc cells where growth and rate of replication is significantly higher (generation time: 1.6 h; Fig. S4) than that of PNIM (generation time: 2.2 h; Fig. S4), cysteine level merely increased to around six fold to 301 µM mg^−1^ protein. It is worth mentioning here that the level of cysteine in LB medium is quite lower than that of proline [18]. The findings thus indicated comparatively preferential regulation of proline metabolism in stressed *Xanthomonas* cells undergoing PCD.

### Inhibition of PCD in *Xanthomonas* by a Proline Analog, a PutA Inhibitor

**Figure 3:**
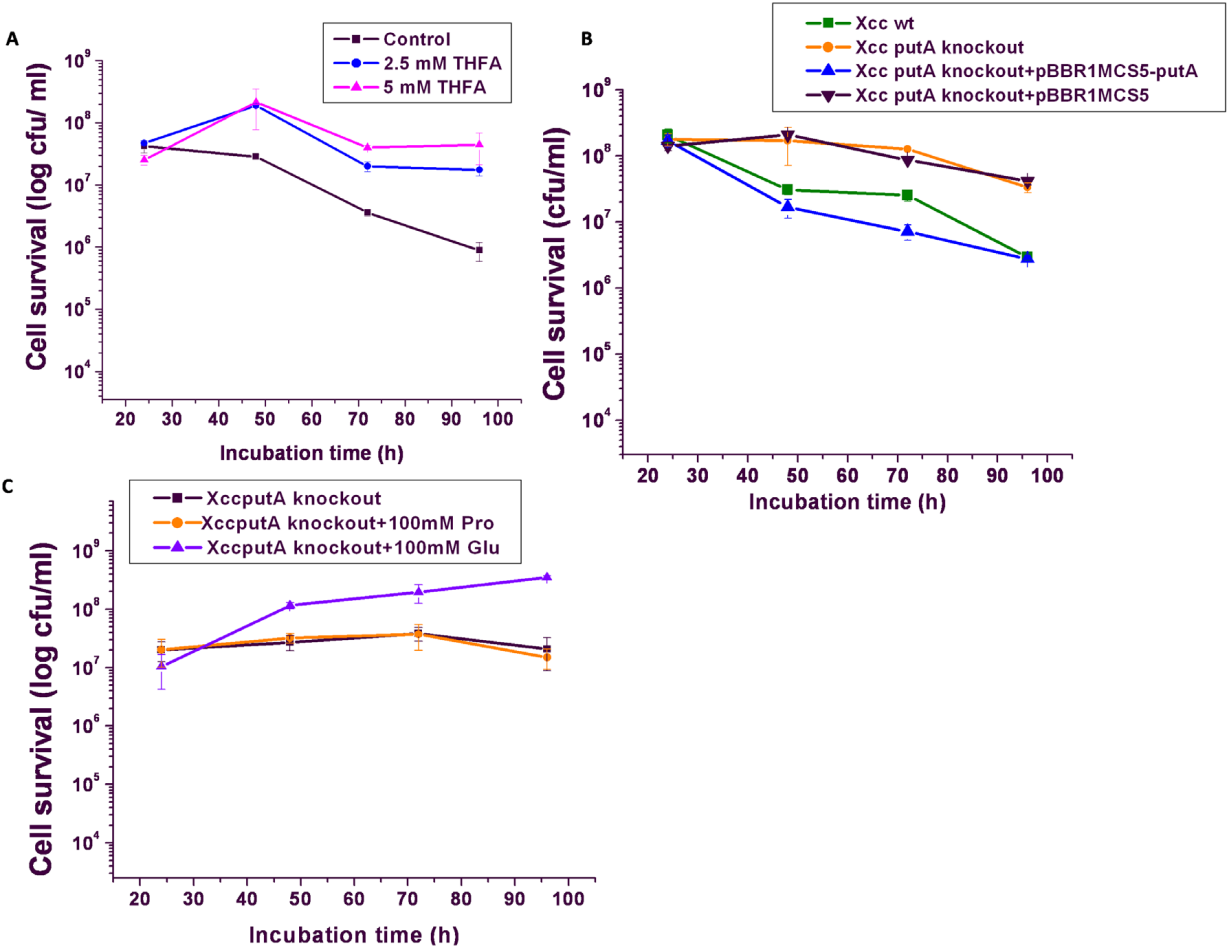
PCD in *Xanthomonas* cells. (**A**) Effect of PutA inhibitor, tetrahydro-2-furoic acid (THFA) on PCD process in Xcc 8004 cells grown in PIM, (**B**) Effect of knocking out of *putA* and its complementation on the PCD profile of Xcc 8004 strains grown in PIM, (**C**) Effect of supplementation of proline and glutamate in PNIM on the growth of Xcc Δ*putA* cells.

The above observations suggested a role of proline metabolism in PCD of this organism which was further revalidated by studying the effect of addition of tetrahydro-2-furoic acid (THFA, ≤5 mM), a competitive inhibitor of PutA (a proline analog) in PIM. Interestingly, the inhibition of PCD was observed in Xcc culture when this PutA inhibitor was added in the medium prior to inoculation, and the extent of PCD inhibition was found to be inhibitor concentration dependent (Fig. 3A). Almost two log cycle increase in the cell viability was observed at 96 h of incubation in the presence of THFA (5 mM). These findings thus confirmed the involvement of PutA activity during PCD of *Xanthomonas* cells in PIM.

### Knocking Out of *putA* Gene in Xcc Abolished PCD and its Complementation Restored the Process

Xcc genome has been sequenced and *putA* gene was found to be present in single copy (NCBI accession number: XC_3907). Knocking out of *putA* resulted in Xcc Δ*putA* strain, which showed an increase in the cell survival in PIM by more than one log cycle (viable plate count 6.3×10^7^ cfu ml^−1^) at 96 h of incubation as compared to the wild type (wt) counterpart (2.5×10^6^ cfu ml^−1^) (Fig. 3B). The viability in Xcc Δ*putA* strain in PNIM was very close to that of wt Xcc cells growing in the same medium (Fig. 3C) and remained unaltered even when PNIM was supplemented with proline (100 mM) unlike that observed for the wt strain (Fig. 3C and 1B respectively). An increase in cell number was observed when Xcc Δ*putA* strain was grown in PNIM supplemented with glutamate (100 mM) (Fig. 3C). The findings thus indicated abolition of PCD upon inhibition of PutA activity in Xcc Δ*putA* strain. PutA activity was also analyzed in Xcc Δ*putA* strain grown in PIM (Fig. 2B). As expected, this activity was found to be completely abolished in Xcc Δ*putA* strain indicating the role of PutA in the above said inhibition of PCD in Xcc Δ*putA* cells growing in PIM.

Further, Xcc Δ*putA* strain was complemented with *putA* gene cloned in a plasmid shuttle vector pBBR1MCS-5 and its viability was monitored in PIM. The PCD phenotype was found to be restored upon complementation with functional PutA. The cell death was 15 fold higher in Xcc Δ*putA* strain carrying pPutA vector (2.75×10^6^ cfu ml^−1^) at 96 h of incubation in PIM compared to the strain carrying the vector without *putA* (4.14×10^7^ cfu ml^−1^) (Fig. 3B). PutA activity was also found to be restored in Xcc Δ*putA* strain upon complementation with functional Xcc PutA and was around three fold higher than the Xcc wt strain growing in PIM (Fig. 2B). However, negligible enzyme activity was detected when this strain was cultured in PNIM (Fig. 2B).

### Status of PCD Specific Markers in Xcc (Wild Type), Xcc Δ*putA* and Xcc Δ*putA-*pPutA Strains

In our earlier studies *Xanthomonas* cells were found to undergo PCD in PIM and displayed certain PCD specific markers such as activation of caspase-3-like protease activity (analyzed by enzyme assay as well as Western blot using polyclonal human caspase-3 antibody), externalization of membrane phosphatidylserine (PS) (assayed using annexinV-FITC labeling), and DNA damage (determined by TUNEL- Terminal deoxynucleotidyl transferase dUTP nick end labeling assay) [1], [2], [3], [4], [5], [21]. The status of these PCD markers was also examined in this study in wild type Xcc, Xcc Δ*putA* and Xcc Δ*putA-* pPutA strains.

### Caspase-3-like Protease Activity Decreased in Xcc Δ*putA* Strain in PIM

**Figure 4:**
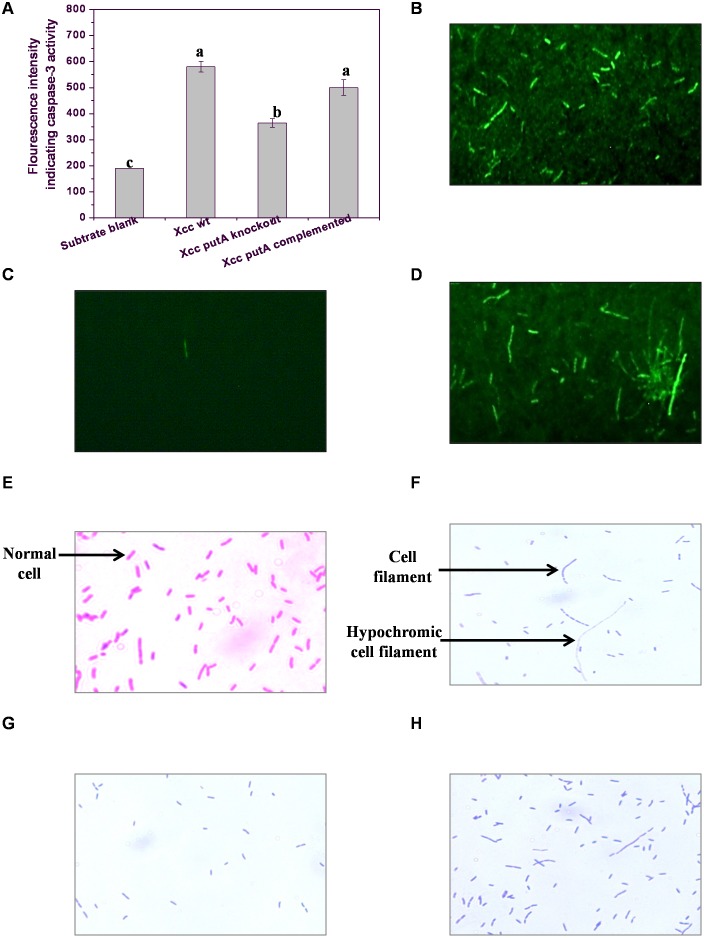
Caspase-3-like protein activity and cell morphology of Xcc strains grown in PIM. (**A**) Caspase-3-like activity in Xcc wt, Xcc Δ*putA*, and Xcc Δ*putA*-pPutA cells in PIM. Different letters on the bars indicate that the means are significantly different at p<0.05. *In situ* labeling with FITC-DEVD-FMK indicating active caspase-3-like protein in (**B**) Xcc wt, (**C**) Xcc Δ*putA* and, (**D**) Xcc Δ*putA*-pPutA cells grown in PIM. Cell morphology of: (**E**) Xcc wt cells in PNIM, (**F**) Xcc wt cells in PIM, (**G**) Xcc Δ*putA* and, (**H**) Xcc Δ*putA*-pPutA cells in PIM.

Caspase-3-like activity which was quantified in terms of fluorescence level of AMC (amino methyl coumarin) released from a synthetic tetrapeptide substrate (Ac-DEVD-AMC) due to protease activity of caspase-3 [22]. Caspase-3-like activity in Xcc Δ*putA* strain while growing in PIM was found to be around 40% less than the wt strain at 24 h of growth in PIM (Fig. 4A). FITC-DEVD-FMK, a fluorescent dye tagged with an irreversible caspase-3 inhibitor (DEVD-FMK) was also used for *in situ* labeling of *Xanthomonas* cells having active caspase-3-like enzyme. Interestingly, Xcc wt and XccΔ*putA-*pPutA cells grown in PIM fluoresced brightly when treated with this dye (Fig. 4 B and D). A negligible number of Xcc Δ*putA* cells fluoresced when treated with FITC-DEVD-FMK (Fig. 4C). Notably, cells showing caspase activity were mostly found to be filamented. Cell filamentation was also observed in PIM growing cells by monochrome (crystal violet) staining. Some hypochromic cell filaments were also observed indicating loss of membrane integrity (Fig. 4F). On the contrary, *Xanthomonas* cells grown in PNIM did not display any significant morphological change even after 72 h of incubation (Fig. 4E). Cell filamentation has been earlier reported in *E.coli* cells exposed to genotoxic agents as a manifestation of SOS response [23]. The current findings thus endorse our earlier report indicating the linkage between bacterial PCD and SOS response [17]. Similar hypothesis has also been proposed in a recent review [5].

### Extent of DNA Damage in Xcc Strains in PIM as Monitored by TUNEL Assay

**Figure 5:**
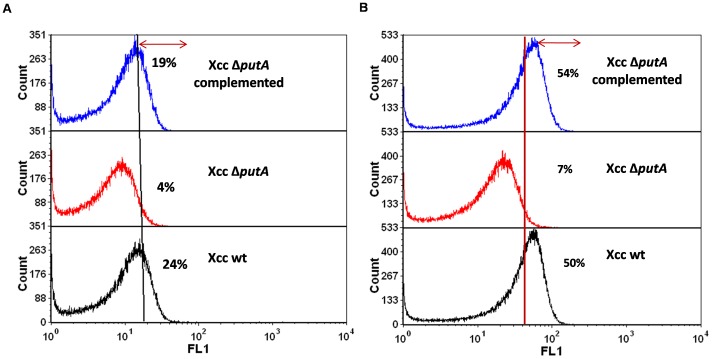
Status of PCD specific markers in Xcc 8004, Xcc Δ*putA* and Xcc *ΔputA*-pPutA cells in PIM. (**A**) TUNEL assay: The gated region (represented by the arrow) depicts the area under the histogram and indicates the percentage of cells labeled by FITC-dUTP which implies DNA damage, (**B**) AnnexinV-FITC assay. The gated region (represented by the arrow) depicts the area under the histogram and indicates the percentage of cells labeled by AnnexinV-FITC.

Activation of caspase-3 activity has been reported to activate CAD (caspase activated DNase) resulting in damage to DNA prior to cell death [24]. With this analogy in this study too, DNA damage was measured in Xcc strains growing in PIM by TUNEL assay. DNA breaks are labeled *in situ* with dUTPs tagged with a fluorophore, fluorescein isothiocyanate (FITC) with the help of an enzyme, Terminal deoxynucleotidyl transferase (TdT) and the extent of labeling which obviously depends upon the extent of DNA damage, is monitored by flow cytometry. This assay has also been used earlier to detect DNA damage in bacteria [25]. Only 4% Xcc Δ*putA* cells were found to be TUNEL positive compared to 24% of Xcc wt and 19% in Xcc Δ*putA* complemented with PutA (Fig. 5A).

### Level of Phosphatidylserine Externalization in Xcc Strains in PIM

Phosphatidylserine (PS) externalization has been reported as an important marker of PCD in various organisms, however, its exact implication remains to be elucidated, particularly in the case of microorganisms including bacteria [26]. It is detected by flow cytometry using AnnexinV-FITC fluorophore. AnnexinV is a 36 kDa Ca^2+^ dependent phospholipid binding protein having high affinity for PS. Only 7% Xcc Δ*putA* cells were found to be AnnexinV-FITC positive as compared to 50% of Xcc wt cells growing in PIM (Fig. 5B).

### Membrane Depolarization in *Xanthomonas* Cells Undergoing PCD in PIM

**Figure 6:**
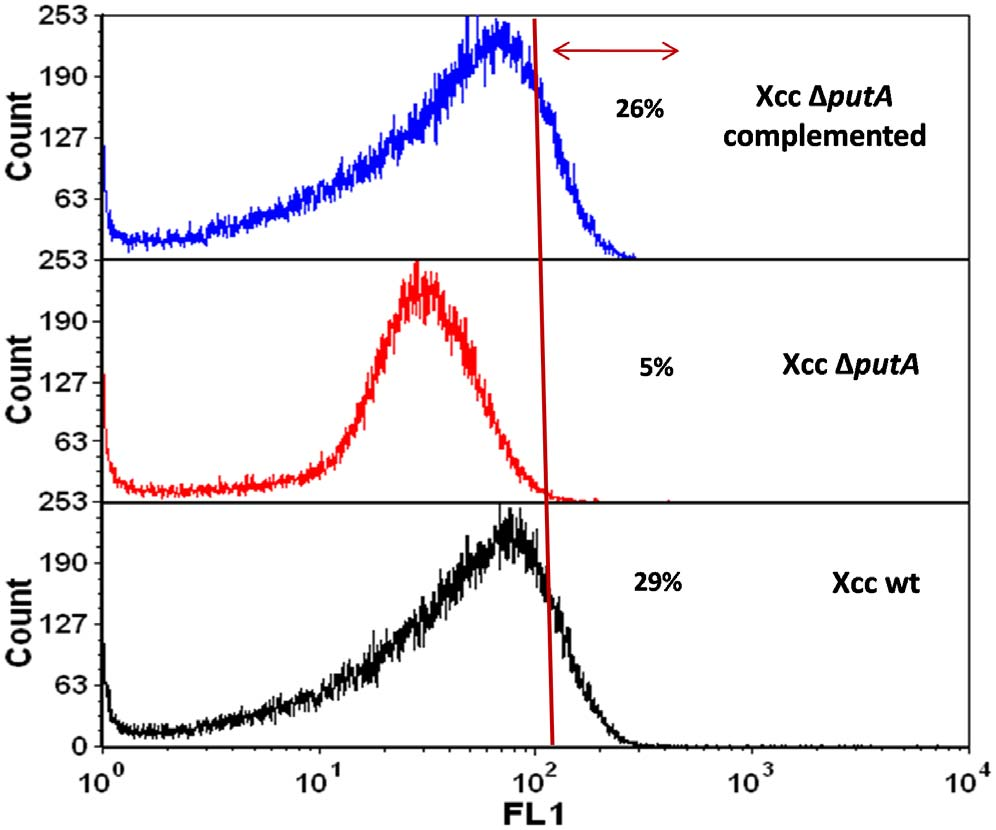
Membrane depolarization in *Xanthomonas* strains in PIM analyzed by flow cytometry. The gated region (represented by the arrow) depicts the area under the histogram and indicates the percentage of depolarized cells.

Recently, membrane depolarization has also been used to monitor PCD in *E.coli* [25]. Membrane depolarization can be monitored by using the membrane potential sensitive carbocyanine dye, DiOC_2_(3) (3, 3′- diethyloxa-carbocyanine iodide). The proportion of depolarized cells was found to be greater in Xcc wt culture (29%) compared to Xcc Δ*putA* (5%) (Fig. 6). In eukaryotes too, mitochondrial membrane depolarization leading to cytochrome c release has been reported as one of the early events occurring during apoptosis [27].

### Possible Mechanism of PutA Activity Associated PCD in *Xanthomonas* Cells Growing in PIM

In our earlier study *Xanthomonas* cells were found to exhibit enhanced level of reactive oxygen species (ROS) generation while undergoing PCD in PIM, and PCD was found to be inhibited in the presence of ROS scavengers [4]. In the previous study, significant elevation in generation of reducing potential (NADH) was observed [4]. Subsequently, a hypothesis was proposed with supportive evidence that there was leakage of electrons from electron transport chain (ETC) during oxidative phosphorylation resulting in excess ROS generation and finally leading to PCD in PIM grown *Xanthomonas* [4].

**Figure 7:**
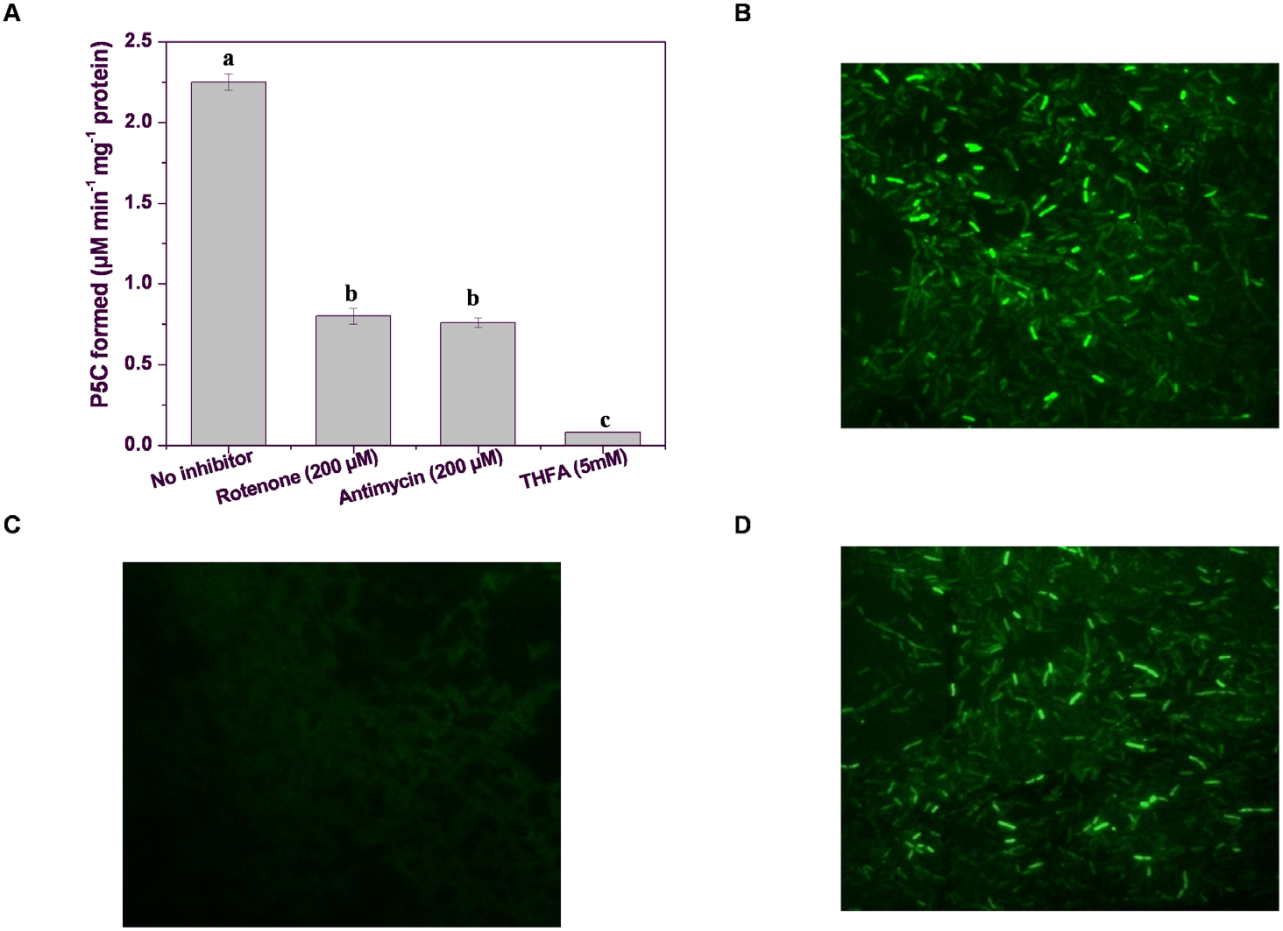
Effect of ETC inhibitors on PutA activity and status of ROS in Xcc strains. (**A**) PutA activity in Xcc 8004 cells in the presence of different inhibitors. Different letters on the bars indicate that the means are significantly different at p<0.05. (**B–D**) Reactive oxygen species (ROS) generation observed by 2′, 7′-dichlorohydrofluorescein-diaceate (H_2_DCFDA) stain in (**B**) Xcc wt, (**C**) Xcc Δ*putA* and, (**D**) Xcc Δ*putA*-pPutA cells in PIM.

In the present study, the possibility of ROS generation was tested during conversion of proline to glutamate by PutA. Proline oxidase (POX), a component of PutA in higher organisms has been reported to be an inner mitochondrial membrane protein that generates electrons during oxidation of proline to glutamate, and subsequent coupled reduction of FAD to FADH_2_. This FADH_2_ transfers electron to ubiquinone (UQ), an electron carrier in ETC. Thus proline can eventually get oxidized to generate ATP as well as superoxide. Though bacteria lack mitochondria, a similar process of electron transfer exists in membrane. Thus, the effect of ETC inhibitors, rotenone and antimycin was studied on Xcc PutA activity. Rotenone inhibits the electron flow from the Fe-S centres of complex I to ubiquinone, whereas, antimycin A inhibits the transfer of electrons from cytochrome b to c_1_ (complex III). Both these inhibitors were found to inhibit the activity of Xcc PutA by 65% (Fig. 7A). Similar inhibitor of complex I (amytal) has been reported as a non- competitive inhibitor of PutA in *E.coli* [28].

The possibility of increased ROS generation upon activation of PutA was examined by monitoring the level of ROS in the Xcc wt, Xcc Δ*putA* and Xcc Δ*putA-*pPutA strains using H_2_DCFDA staining (Fig. 7B, C and D). This observation indicated that PutA was partly involved in generating ROS in *Xanthomonas* cells growing in PIM. The structure and kinetic study of proline dehydrogenase from *Thermus thermophilus* has indicated that this enzyme can directly interact with O_2_, generating superoxide radical [29].

## Discussion

Proline metabolism is distinct from that of primary amino acids and plays a regulatory role in certain physiological conditions [30]. Proline catabolism has been observed to be an important source of energy in some Gram negative bacteria such as *Helicobacter pylori*, *Bradyrhizobium japonicum, Sinorhizobium meliloti,* and *Mycobacterium smegmatis* during stress [31], [32], [33], [34], [35]. PutA is significantly upregulated in *Mycobacterium smegmatis* and is preferentially used as an electron donor to the respiratory chain during energy limiting conditions [35]. The role of PutA during metabolic stress induced cell death in *Xanthomonas* was investigated in this study. A significant increase in the intracellular proline levels and PutA activity was observed in PCD inducing medium or PIM (LB broth) implying the involvement of this enzyme in the programmed cell death (PCD) of *Xanthomonas* (Fig. 2). LB broth used in this study is predominantly composed of casein digest and yeast extract both of which are rich in several amino acids and peptides [18]. As a result, the microorganism has to use them both as carbon and nitrogen source. During such growth conditions certain metabolic pathways can be preferentially upregulated over others. High intracellular levels of proline in *Xanthomonas* cells can be attributed to abundant free proline and peptides present in PIM. High proline levels have been reported to induce PutA activity as well [36]. A good correlation was found between intracellular proline levels and PutA activity in Xcc wt cells cultured in PIM (Fig. 2). Addition of proline in PNIM also resulted in induction of PCD in *Xanthomonas campestris* cells (Fig. 1). However, the presence of glutamate in PNIM did not induce PCD, instead it supported the growth of *Xanthomonas* (Fig. 1). This observation is consistent with our earlier study where α-ketoglutarate when added in PNIM was not found to induce PCD in *Xanthomonas* [3]. Glutamate and glutamine are important amino acids for bacterial metabolism [37]. Glutamate participates in both catabolism and anabolism. It can be deaminated to form α-ketoglutarate, a TCA intermediate. Glutamate accounts for ∼88% of cellular nitrogen and is required for the biosynthesis of purines, pyrimidines and amino sugars [37]. Hence, when starch minimal medium (PCD non–inducing medium or PNIM) was supplemented with excess glutamate, it provided a ready source of nitrogen favoring anabolic events as it was observed to accelerate *Xanthomonas* growth. In a recent study, α-ketoglutarate has been shown to contribute to a variety of metabolic processes including TCA cycle, biogenesis of several amino acids, carnitine biosynthesis and as a cofactor in several dioxygenases [38]. It is also reported to be involved in the detoxification of ROS during oxidative stress [38]. Due to this possible reason, addition of glutamate or α-ketoglutarate in PNIM was not found to induce PCD in *Xanthomonas*. The exact reason for this observation is still unresolved. Surprisingly, other TCA intermediates like pyruvate, citrate and malate were found to induce PCD in an earlier study from this laboratory [3]. The differential role of these TCA intermediates with respect to induction of PCD process indicates the complexity of the process and is subject to further study.

The presence of proline and glutamate together in PNIM also induced cell death indicating proline to have a major role in cellular regulation probably because proline to glutamate conversion is accompanied with enhanced ROS generation leading to oxidative stress. Further, the role of PutA was revalidated by the observed inhibition of cell death in the presence of THFA, a competitive inhibitor of PutA (Fig. 3A). These results indicated that it is not excess proline but its increased oxidation by PutA that caused cell death.

Paradoxically, proline has been reported to act as an osmoprotectant in certain bacteria growing under osmotic stress [39], [40]. Besides bacteria, proline has been shown to protect fungi, plants, and mammalian cells against oxidative stress [41]. Proline accumulation has also been reported in plants during conditions of drought, salinity, intense light, UV irradiation, heavy metals, oxidative stress, and biotic stresses [42], [43].

Proline porter I and PutA regulate the catabolism of proline in *E.coli* [44]. They are induced when proline is provided as a carbon or nitrogen source in the environment. *E.coli* PutA regulates the transcription of *putA* and *putP* (Na^+^/proline symporter) genes, and it switches its intracellular location and function by sensing the environmental proline levels. When the intracellular proline levels are low, it binds to DNA and represses the transcription of *put* genes. Conversely, when proline is available to the cell, PutA binds to the inner membrane and catalyzes the oxidation of proline to glutamate [45]. However, in *Salmonella, Klebsiella* and *Vibrio* the *putA* gene expression is regulated by c-AMP receptor protein [46], [47], [48]. *Xanthomonas* PutA has not been studied yet and the regulation of proline metabolism in this bacterium is not fully understood.

To confirm the role of PutA in PCD of *Xanthomona*s, a *putA* knockout of *Xanthomonas campestris* pv. *campestris* (Xcc) was constructed. The post-exponential death phase observed prominently in the wt strain grown in PIM was found to be absent in Xcc Δ*putA* (Fig. 3B). In these cells, proline was around 1.5 fold higher than the wt cells (Fig. 2A). The observation further confirms that the high level of proline alone does not cause death, rather it is the enhanced metabolism of proline by PutA which induces this event as observed by the increased viability of Xcc Δ*putA* cells in PIM (Fig. 3B). Further, complementing Xcc Δ*putA* strain with functional PutA resulted in cell death when this strain was grown in PIM (Fig. 3B). The markers of apoptosis like externalization of PS and loss of membrane potential were also observed in *Xanthomonas* cells undergoing PCD (Fig. 5B and 6). The fraction of AnnexinV positive cells was significantly less in Xcc Δ*putA* culture (Fig. 5B). PS externalization has been reported as the hallmark of PCD in various systems including *E.coli*, *Saccharomyces* and *Aspergillus* [26], [49], [50].

In a previous study ROS was found to cause PCD in *Xanthomonas* when cells were cultured in PIM and addition of certain antioxidants was found to inhibit this process [4], [5]. The extent of DNA damage and the level of ROS were found to be lower in Xcc Δ*putA* compared to the wt strain implying that PutA is involved in ROS generation leading to DNA damage and eventually cell death (Fig. 5A, 7B and C). *Helicobacter* PutA has also been reported to have high reactivity with molecular oxygen leading to the formation of ROS [51]. Interestingly, structural biology studies have revealed that proline dehydrogenase of the bacterium *Thermus thermophilus* directly interacts with oxygen to produce superoxide radical [29]. The flavin adenine dinucleotide (FAD) cofactor of this enzyme is accessible to dissolved oxygen allowing the direct reduction of O_2_ to superoxide. Hence, the electrons from proline can be used to generate ROS. Recently, it has been shown that proline dehydrogenase of *Arabidopsis* is involved in ROS formation during the hypersensitive response [43], [52]. Similarly, human POX contributes to apoptosis by generation of ROS (mainly superoxide) either directly by interacting with oxygen at the enzyme active site or indirectly by increasing the electron flux in the electron transport chain [53]. Interestingly, the recently discovered P5C–proline cycle can deliver electrons to mitochondrial electron transport without producing glutamate and, under certain conditions, can generate more ROS in the mitochondria [42], [54]. Proline catabolism is, therefore, an important regulator of cellular ROS balance and can influence numerous additional regulatory pathways. Proline metabolism has been reported to influence cellular ATP and NADPH/NADP+ ratio during oxidative and nutrient stress in animal cell lines [55]. Similarly, *Xanthomonas* PutA activity was found to be linked to ETC (Fig. 7A) suggesting that PutA is also involved in regulating the redox homeostasis of the cell.

Additionally, cell filamentation was observed in PIM grown *Xanthomonas* cells exhibiting caspase-3-like activity during *in situ* labeling with cell permeable caspase-3 inhibitor (Fig. 4B). Among different possible explanations for cell filamentation, one could be due to ROS mediated DNA damage leading to upregulation of error prone repair pathway like SOS response in different bacteria. The induction of SOS response depends upon the extent and nature of DNA damage. The activation of caspase-3-like protein and SOS response in *E.coli* has been reported in an earlier study from this laboratory [17].

POX is reported to play an important role in cancer, apoptosis and schizophrenia in humans [56]. It has been found to be one of the 14 genes to be induced more than 10 fold by p53 and has been termed as p53-induced gene 6 (PIG6) [36]. POX is regarded as a tumor suppressor protein and any anomaly in its functioning results in cancer [57]. POX activation in higher systems has been reported to induce both intrinsic and extrinsic pathways of apoptosis by regulating the redox homeostasis of the cell and has been observed to activate caspase-3, 8 and 9 [58]. Overexpression of POX leads to apoptotic cell death in several cancer cell types [57], [58], [59]. Its role has been established in eukaryotic apoptosis and is considered as an important protein for preventing initiation of cancer.

This study provides evidence of involvement of proline oxidase in the observed programmed cell death of *Xanthomonas.* The proline oxidase linked leakage of electrons from the electron transport chain caused ROS generation and the resultant activation of caspase-3-like protein led to cell death. The findings are quite similar to the events observed in higher organisms indicating an evolutionarily conserved role of this protein in PCD.

## Supporting Information

Figure S1 **Schematic representation of insertional mutagenesis of Xcc8004** ***putA*** **using pKNOCK vector.**
(TIF)

Figure S2 **Gene organization of Xcc** ***putA*** **and its upstream non coding sequence included in** ***putA*** **complementation construct.** (**A**) Organization of *putA* (NCBI gene ID: 3379526) in Xcc genome, (**B**) The sequence of upstream non coding promoter containing region (319 bp; source: NCBI database) of Xcc *putA* included in *putA* complementation construct. BPROM software was used for promoter prediction (−10 and −35 box). FP and RP indicate the sites for forward and reverse primers respectively, used for PCR amplification of *putA* along with its promoter.
(TIF)

Figure S3 **pBBR1MCS5 vector map depicting cloned** ***putA*** **(full length) along with its promoter region.**
(TIF)

Figure S4 **Growth curve of** ***Xanthomonas campestris*** **pv.** ***campestris*** **strain 8004 in PCD inducing medium (PIM) and PCD non-inducing medium (PNIM).**
(TIF)

## References

[1] Gautam S, Sharma A (2002a) Rapid cell death in *Xanthomonas campestris* pv. *glycines* . J Gen Appl Microbiol 48: 67–76. 12469302 10.2323/jgam.48.67

[2] Gautam S, Sharma A (2002b) Involvement of caspase-3-like protein in rapid cell death of *Xanthomonas.* . Mol Microbiol 44: 393–401. 11972778 10.1046/j.1365-2958.2002.02837.x

[3] Raju KK, Gautam S, Sharma A (2006) Molecules involved in the modulation of rapid cell death in *Xanthomonas* . J Bacteriol 188: 5408–5416. 16855230 10.1128/JB.00056-06 PMC1540037

[4] Wadhawan S, Gautam S, Sharma A (2010) Metabolic stress-induced programmed cell death in *Xanthomonas* . FEMS Microbiol Lett 312: 176–183. 20958788 10.1111/j.1574-6968.2010.02114.x

[5] Bayles KW (2014) Bacterial programmed cell death: making sense of a paradox. Nat Rev Microbiol 12: 63–69. 24336185 10.1038/nrmicro3136 PMC4422510

[6] Asplund-Samuelsson J, Bergman B, Larsson J (2012) Prokaryotic caspase homologs: phylogenetic patterns and functional characteristics reveal considerable diversity. PLoS ONE 7: e49888. 23185476 10.1371/journal.pone.0049888 PMC3501461

[7] Zhang M, White TA, Schuermann JP, Baban BA, Becker DF, et al (2004) Structures of the *Escherichia coli* PutA proline dehydrogenase domain in complex with competitive inhibitors. Biochemistry 43: 12539–12548. 15449943 10.1021/bi048737e PMC3727243

[8] Phang JM, Liu W, Hancock C, Christian KJ (2012) The proline regulatory axis and cancer. Front Oncol 2: 1–12. 10.3389/fonc.2012.00060 PMC3380417 22737668

[9] Bates LS, Waldren RP, Teare ID (1973) Rapid determination of free proline for water stress studies. Plant soil 39: 205–207.

[10] Gaitonde MK (1967) A spectrophotometric method for the direct determination of cysteine in the presence of other naturally occurring amino acids. Biochem J 104: 627–633. 6048802 10.1042/bj1040627 PMC1270629

[11] Dendinger S, Brill WJ (1970) Regulation of proline degradation in *Salmonella typhimurium.* . J Bacteriol 103: 144–152. 4912518 10.1128/jb.103.1.144-152.1970 PMC248050

[12] Lowry OH, Rosebrough NJ, Farr AL, Randall RJ (1951) Protein measurement with the Folin phenol reagent. J Biol Chem 193: 265–275. 14907713

[13] Alexeyev MF (1999) The pKNOCK series of broad-host-range mobilizable suicide vectors for gene knockout and targeted DNA insertion into the chromosome of gram-negative bacteria. Biotechniques 26: 824–826. 10337469 10.2144/99265bm05

[14] Ratnakar PV, Mohanty BK, Lobert M, Bastial D (1996) The replication initiator protein pi of the plasmid R6K specifically interacts with the host-encoded helicase DnaB. PNAS 93: 5522–5526. 8643608 10.1073/pnas.93.11.5522 PMC39279

[15] Sambrook J, Fritsch EF, Maniatis T (1989) Molecular cloning. Cold Spring Harbor Laboratory Press 1.75 p.

[16] Kovach ME, Elzer PH, Hill DS, Robertson GT, Farris MA, et al (1995) Four new derivatives of the broad-host-range cloning vector pBBR1MCS, carrying different antibiotic-resistance cassettes. Gene 166: 175–176. 8529885 10.1016/0378-1119(95)00584-1

[17] Wadhawan S, Gautam S, Sharma A (2013) A component of gamma radiation induced cell death in *E. coli* is programmed and interlinked with activation of caspase-3 and SOS response. Arch Microbiol 195: 545–557. 23807199 10.1007/s00203-013-0906-6

[18] Sezonov G, Joseleau-Petit D, D’Ari R (2007) *Escherichia coli* physiology in luria-bertani broth. J Bacteriol 189: 8746–8749. 17905994 10.1128/JB.01368-07 PMC2168924

[19] BD Bionutrients technical manual (2006) Becton, Dickinson and Company, USA. 53 p.

[20] Liu W, Phang JM (2012) Proline dehydrogenase (oxidase) in cancer. Biofactors 38: 398–406. 22886911 10.1002/biof.1036 PMC7479541

[21] Rice KC, Bayles KW (2003) Death’s toolbox: examining the molecular components of bacterial programmed cell death. Mol Microbiol 50: 729–738. 14617136 10.1046/j.1365-2958.2003.t01-1-03720.x

[22] Elmore S (2007) Apoptosis: a review of programmed cell death. Toxicol Pathol 35: 495–516. 17562483 10.1080/01926230701320337 PMC2117903

[23] Janion C (2008) Inducible SOS response system of DNA repair and mutagenesis in *Escherichia coli.* . Int J Biol Sci 4: 338–344. 18825275 10.7150/ijbs.4.338 PMC2556049

[24] Earnshaw WC, Martins LM, Kaufmann SH (1999) Mammalian caspases: structure, activation, substrates, and functions during apoptosis. Annu Rev Biochem 68: 383–424. 10872455 10.1146/annurev.biochem.68.1.383

[25] Erental A, Sharon I, Engelberg-Kulka H (2012) Two programmed cell death systems in *Escherichia coli*: an apoptotic-like death is inhibited by the *mazEF*-mediated death pathway. PLoS Biol 10: e1001281. 22412352 10.1371/journal.pbio.1001281 PMC3295820

[26] Dwyer DJ, Camacho DM, Kohanski MA, Callura JM, Collins JJ (2012) Antibiotic-induced bacterial cell death exhibits physiological and biochemical hallmarks of apoptosis. Mol Cell 46: 561–572. 22633370 10.1016/j.molcel.2012.04.027 PMC3710583

[27] Gottlieb E, Armour SM, Harris MH, Thompson CB (2003) Mitochondrial membrane potential regulates matrix configuration and cytochrome c release during apoptosis. Cell Death Differ 10: 709–717. 12761579 10.1038/sj.cdd.4401231

[28] Abrahamson JL, Baker LG, Stephenson JT, Wood JM (1983) Proline dehydrogenase from *Escherichia coli* K12: properties of the membrane-associated enzyme. Eur J Biochem 134: 77–82. 6305659 10.1111/j.1432-1033.1983.tb07533.x

[29] White TA, Krishnan N, Becker DF, Tanner JJ (2007) Structure and kinetics of monofunctional proline dehydrogenase from *Thermus thermophilus* . J Biol Chem 282: 14316–14327. 17344208 10.1074/jbc.M700912200 PMC2708979

[30] Phang JM, Donald SP, Pandhare J, Liu Y (2008) The metabolism of proline, a stress substrate, modulates carcinogenic pathways. Amino Acids 35: 681–690. 18401543 10.1007/s00726-008-0063-4

[31] Kohl DH, Straub PF, Shearer G (1994) Does proline play a special role in bacteroid metabolism? Plant Cell Environ 17: 1257–1262.

[32] Dillewijn PV, Soto MJ, Villadas PJ, Toro N (2001) Construction and environmental release of a *Sinorhizobium meliloti* strain genetically modified to be more competitive for alfalfa nodulation. Appl Environ Microbiol 67: 3860–3865. 11525978 10.1128/AEM.67.9.3860-3865.2001 PMC93102

[33] Nagata K, Nagata Y, Sato T, Fujino MA, Nakajima K, et al (2003) L-Serine, D- and L-proline and alanine as respiratory substrates of *Helicobacter pylori*: correlation between in vitro and in vivo amino acid levels. Microbiology 149: 2023–2030. 12904542 10.1099/mic.0.26203-0

[34] Curtis J, Shearer G, Kohl DH (2004) Bacteroid proline catabolism affects N_2_ fixation rate of drought-stressed soybeans. Plant Physiol 136: 3313–3318. 15448193 10.1104/pp.104.044024 PMC523390

[35] Berney M, Cook GM (2010) Unique flexibility in energy metabolism allows *Mycobacteria* to combat starvation and hypoxia. PLoS ONE 5: e8614. 20062806 10.1371/journal.pone.0008614 PMC2799521

[36] Donald SP, Sun XY, Hu CA, Yu J, Mei JM, et al (2001) Proline Oxidase, encoded by p53-induced gene-6, catalyzes the generation of proline-dependent reactive oxygen species. Cancer Res 61: 1810–1815. 11280728

[37] Yan D (2007) Protection of glutamate pool concentration in enteric bacteria. PNAS 104: 9475–9480. 17517610 10.1073/pnas.0703360104 PMC1890519

[38] Mailloux RJ, Singh R, Brewer G, Auger C, Lemire J, et al (2009) Alpha-ketoglutarate dehydrogenase and glutamate dehydrogenase work in tandem to modulate the antioxidant alpha-ketoglutarate during oxidative stress in *Pseudomonas fluorescens* . J Bacteriol 191: 3804–3810. 19376872 10.1128/JB.00046-09 PMC2698380

[39] Anderson CB, Witter LD (1982) Glutamine and proline accumulation by *Staphylococcus aureus* with reduction in water activity. Appl Environ Microbiol 43: 1501–1503. 7103493 10.1128/aem.43.6.1501-1503.1982 PMC244260

[40] Wood JM, Bremer E, Kraemer LNC, Poolman B, Heide TVD, et al (2001) Osmosensing and osmoregulatory compatible solute accumulation by bacteria. Comp Biochem Physiol A Mol Integr Physiol 130: 437–460. 11913457 10.1016/s1095-6433(01)00442-1

[41] Krishnan N, Dickman MB, Becker DF (2008) Proline modulates the intracellular redox environment and protects mammalian cells against oxidative stress. Free Radic Biol Med 44: 671–681. 18036351 10.1016/j.freeradbiomed.2007.10.054 PMC2268104

[42] Szabados L, Savoure A (2010) Proline: a multifunctional amino acid. Trends Plant Sci 15: 89–97. 20036181 10.1016/j.tplants.2009.11.009

[43] Cecchini NM, Monteoliva MI, Alvarez ME (2011) Proline dehydrogenase contributes to pathogen defense in *Arabidopsis* . Plant Physiol 155: 1947–1959. 21311034 10.1104/pp.110.167163 PMC3091113

[44] Milner JL, McClellan DJ, Wood JM (1987) Factors reducing and promoting the effectiveness of proline as an osmoprotectant in *Escherichia coli* K12. J Gen Microbiol 133: 1851–1860. 3312483 10.1099/00221287-133-7-1851

[45] Zhou Y, Zhu W, Bellur PS, Rewinkel D, Becker DF (2008) Direct linking of metabolism and gene expression in the proline utilization A protein from *Escherichia coli* . Amino Acids 4: 711–718. 10.1007/s00726-008-0053-6 PMC2666929 18324349

[46] Hahn DR, Maloy SR (1986) Regulation of the *put* operon in *Salmonella typhimurium*: characterization of promoter and operator mutations. Genetics 114: 687–703. 3539694 10.1093/genetics/114.3.687 PMC1203008

[47] Chen LM, Maloy S (1991) Regulation of proline utilization in enteric bacteria: cloning and characterization of the *Klebsiella put* control region. J Bacteriol 173: 783–790. 1987164 10.1128/jb.173.2.783-790.1991 PMC207072

[48] Lee JH, Park NY, Lee MH, Choi SH (2003) Characterization of the *Vibrio vulnificus putAP* operon, encoding proline dehydrogenase and proline permease, and its differential expression in response to osmotic stress. J Bacteriol 185: 3842–3852. 12813078 10.1128/JB.185.13.3842-3852.2003 PMC161561

[49] Ludovico P, Rodrigues F, Almeida A, Silva MT, Barrientos A, et al (2002) Cytochrome c release and mitochondria involvement in programmed cell death induced by acetic acid in *Saccharomyces cerevisiae* . Mol Biol Cell 13: 2598–2606. 12181332 10.1091/mbc.E01-12-0161 PMC117928

[50] Amin S, Mousavi A, Robson GD (2004) Oxidative and amphotericin B-mediated cell death in the opportunistic pathogen *Aspergillus fumigatus* is associated with an apoptotic-like phenotype. Microbiology 150: 1937–1945. 15184579 10.1099/mic.0.26830-0

[51] Krishnan N, Becker DF (2006) Oxygen reactivity of PutA from *Helicobacter* species and proline-linked oxidative stress. J Bacteriol 188: 1227–1235. 16452403 10.1128/JB.188.4.1227-1235.2006 PMC1367249

[52] Cecchini NM, Monteoliva MI, Alvarez ME (2011) Proline dehydrogenase is a positive regulator of cell death in different kingdoms. Plant Signal Behav 6: 1195–1197. 21757996 10.4161/psb.6.8.15791 PMC3260720

[53] Wanduragala S, Sanyal N, Liang X, Becker DF (2010) Purification and characterization of Put1p from *Saccharomyces cerevisiae.* . Arch Biochem Biophys 498: 136–142. 20450881 10.1016/j.abb.2010.04.020 PMC2880193

[54] Miller G, Honig A, Stein H, Suzuki N, Mittler R, et al (2009) Unraveling delta1-pyrroline-5-carboxylate-proline cycle in plants by uncoupled expression of proline oxidation enzymes. J Biol Chem 25: 26482–26492. 10.1074/jbc.M109.009340 PMC2785336 19635803

[55] Natarajan SK, Becker DF (2012) Role of apoptosis-inducing factor, proline dehydrogenase, and NADPH oxidase in apoptosis and oxidative stress. Cell Health Cytoskelet 4: 11–27. 10.2147/CHC.S4955 PMC3351110 22593641

[56] Luo M, Arentson BW, Srivastava D, Becker DF, Tanner JJ (2012) Crystal structures and kinetics of monofunctional proline dehydrogenase provide insight into substrate recognition and conformational changes associated with flavin reduction and product release. Biochemistry 51: 10099–10108. 23151026 10.1021/bi301312f PMC3525754

[57] Liu Y, Borchert GL, Donald SP, Diwan BA, Anver M, et al (2009) Proline oxidase functions as a mitochondrial tumor suppressor in human cancers. Cancer Res 69: 6414–6422. 19654292 10.1158/0008-5472.CAN-09-1223 PMC4287397

[58] Liu Y, Borchert GL, Surazynski A, Hu CA, Phang JM (2006) Proline oxidase activates both intrinsic and extrinsic pathways for apoptosis: The role of ROS/superoxides, NFAT and MEK/ERK signaling. Oncogene 25: 5640–5647. 16619034 10.1038/sj.onc.1209564

[59] Maxwell SA, Rivera A (2003) Proline oxidase induces apoptosis in tumor cells, and its expression is frequently absent or reduced in renal carcinomas. J Biol Chem 278: 9784–9789. 12514185 10.1074/jbc.M210012200

[60] Turner P, Barber C, Daniels M (1984) Behaviour of the transposons Tn5 and Tn7 in *Xanthomonas campestris* pv. *campestris* . Mol Gen Genet 195: 101–107.

