# Supplementary figures and images for: Involvement of Proline Oxidase (PutA) in Programmed Cell Death of Xanthomonas
Source: PLoS One. 2014 May 1;9(5):e96423. doi: 10.1371/journal.pone.0096423 (PMC4006831; doi:10.1371/journal.pone.0096423)

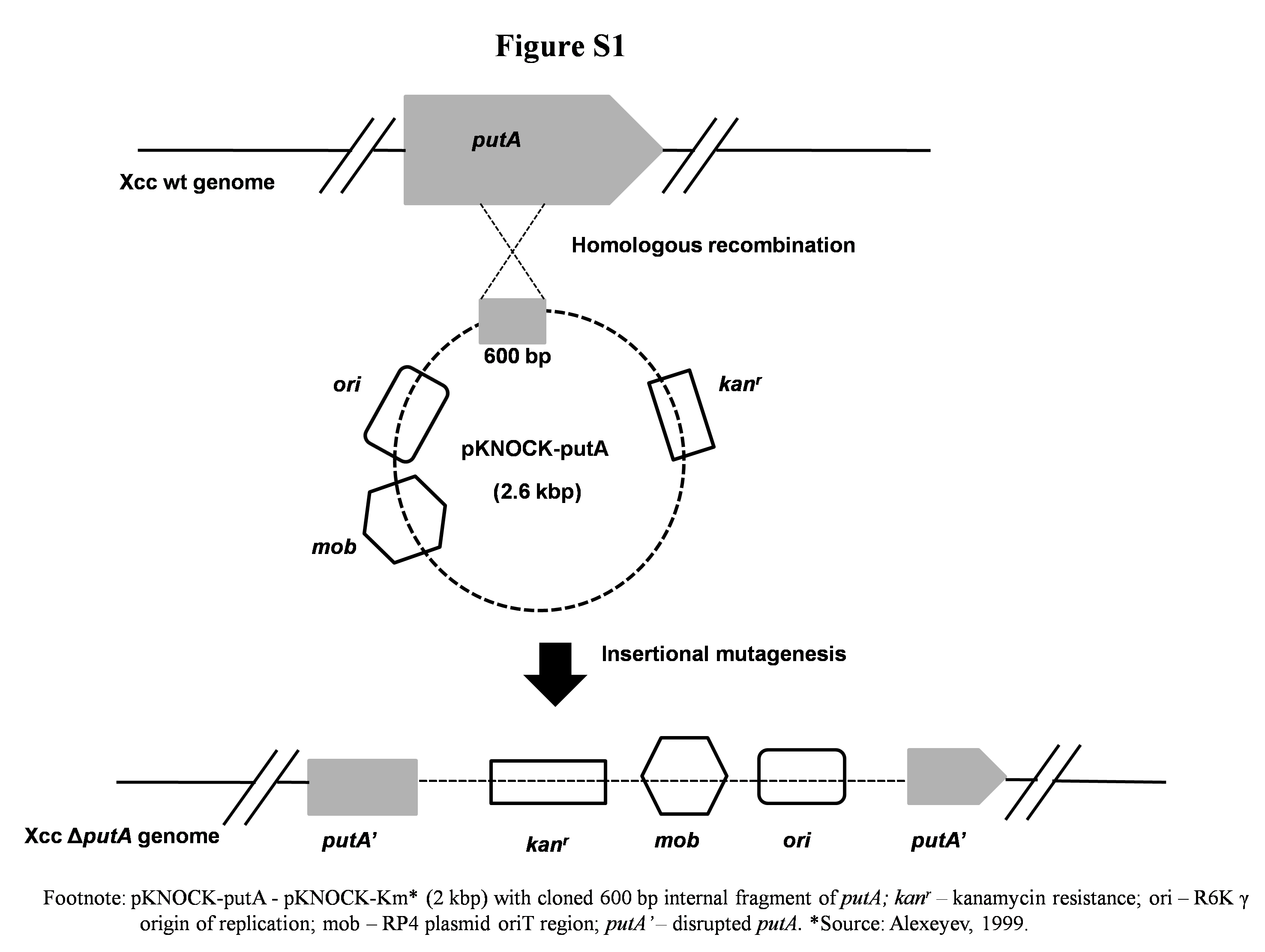

Supplement: Figure S1 — Schematic representation of insertional mutagenesis of Xcc8004 putA using pKNOCK vector. (TIF) [file pone.0096423.s001.tif]

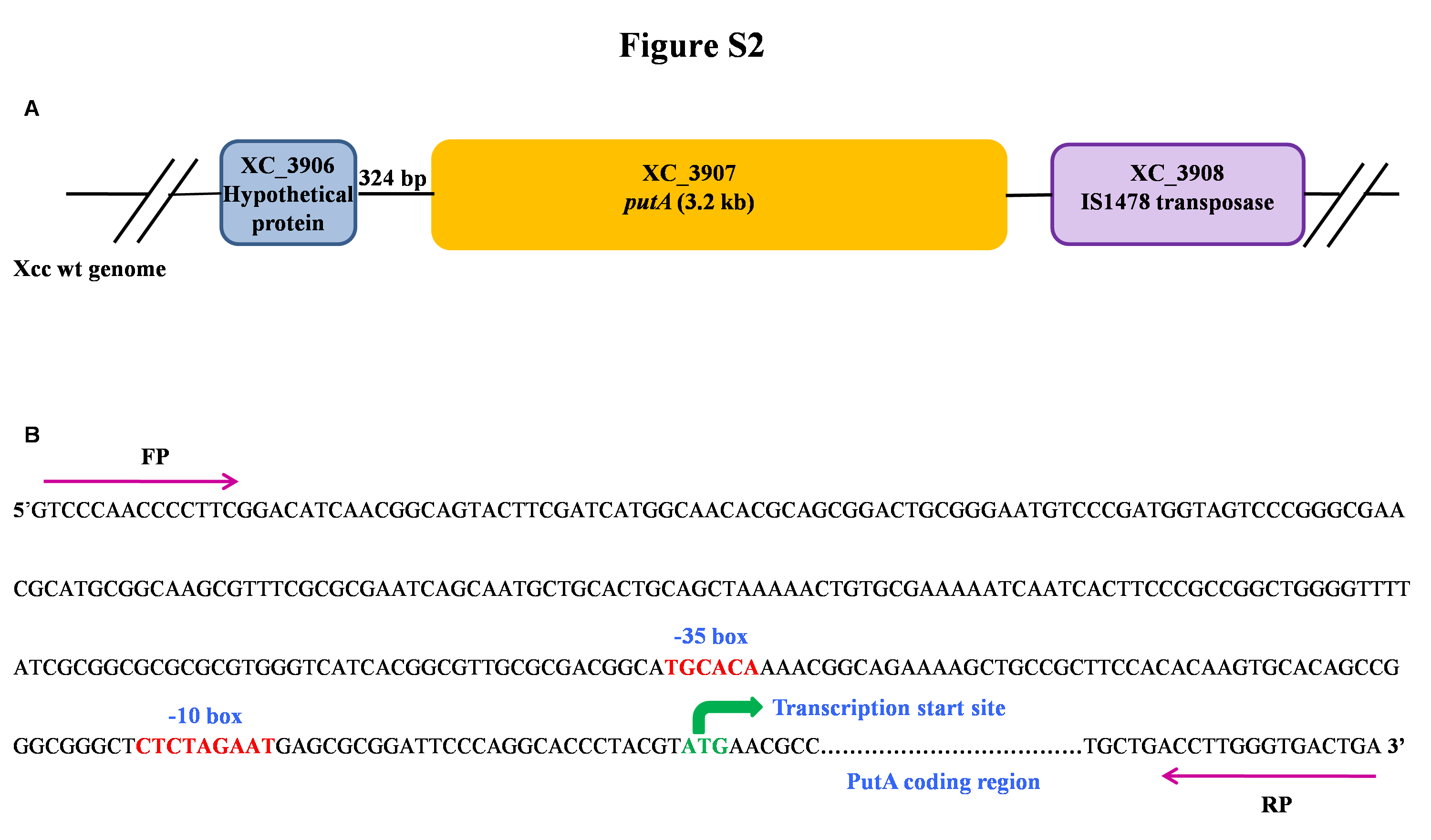

Supplement: Figure S2 — Gene organization of Xcc putA and its upstream non coding sequence included in putA complementation construct. (A) Organization of putA (NCBI gene ID: 3379526) in Xcc genome, (B) The sequence of upstream non coding promoter containing region (319 bp; source: NCBI database) of Xcc putA included in putA complementation construct. BPROM software was used for promoter prediction (−10 and −35 box). FP and RP indicate the sites for forward and reverse primers respectively, used for PCR amplification of putA along with its promoter. (TIF) [file pone.0096423.s002.tif]

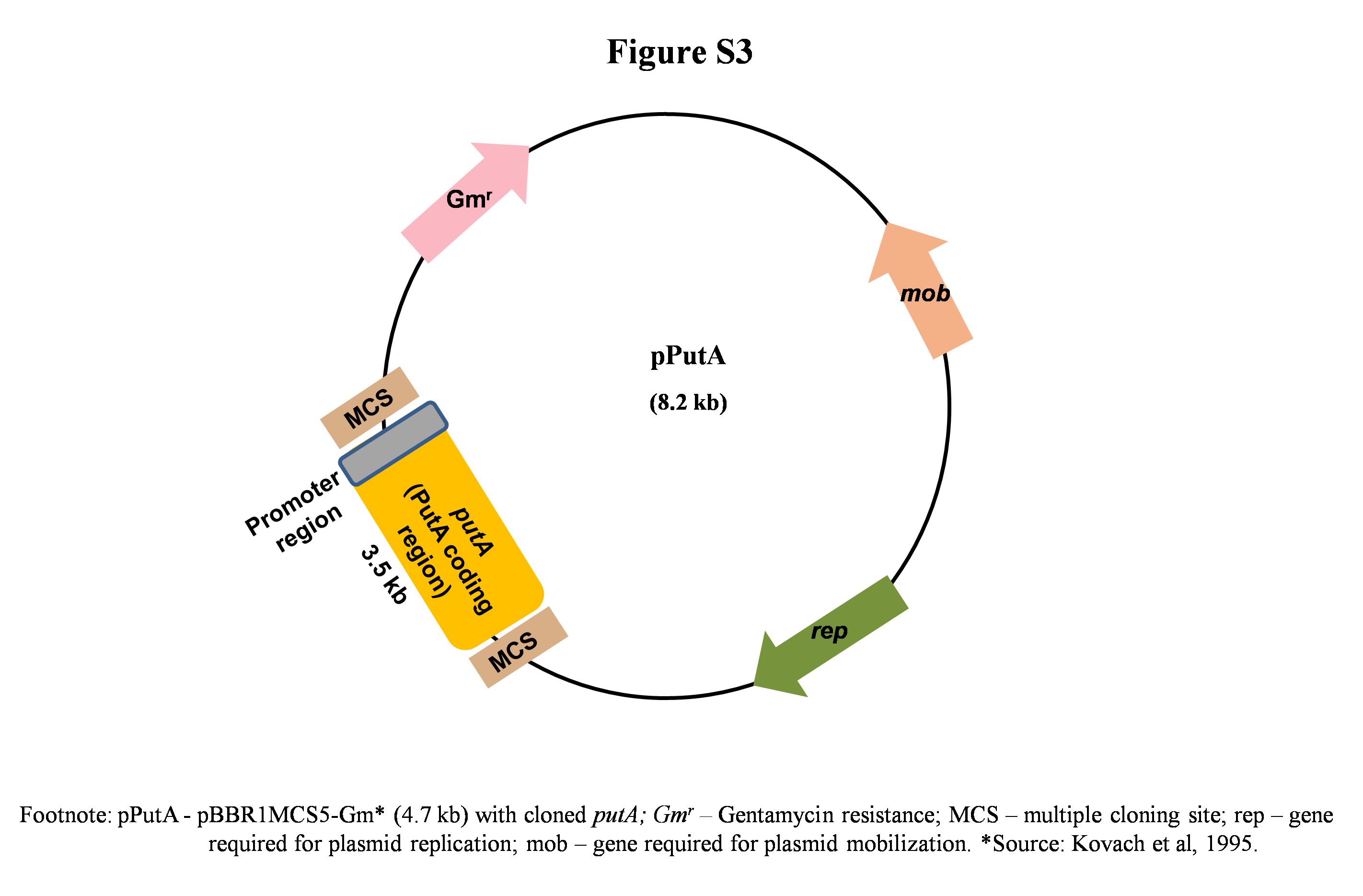

Supplement: Figure S3 — pBBR1MCS5 vector map depicting cloned putA (full length) along with its promoter region. (TIF) [file pone.0096423.s003.tif]

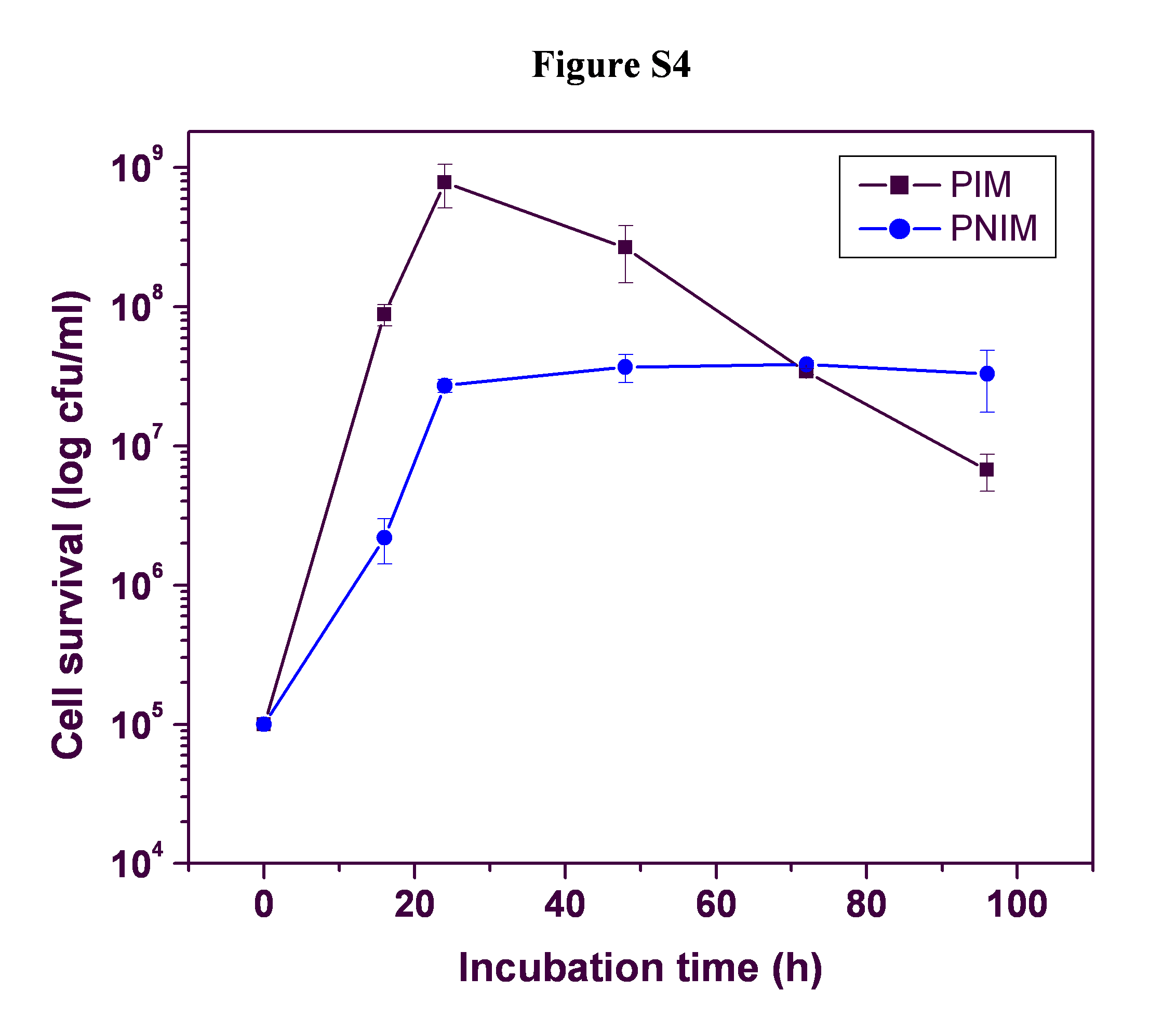

Supplement: Figure S4 — Growth curve of Xanthomonas campestris pv. campestris strain 8004 in PCD inducing medium (PIM) and PCD non-inducing medium (PNIM). (TIF) [file pone.0096423.s004.tif]
